# Development of a Flexible Non-Metal Electrode for Cell Stimulation and Recording

**DOI:** 10.3390/s16101613

**Published:** 2016-09-29

**Authors:** Cihun-Siyong Alex Gong, Wun-Jia Syu, Kin Fong Lei, Yih-Shiou Hwang

**Affiliations:** 1 Department of Electrical Engineering, School of Electrical and Computer Engineering, College of Engineering, Chang Gung University, Taoyuan 33302, Taiwan; alex.mlead@gmail.com; 2 Portable Energy System Group, Green Technology Research Center, College of Engineering, Chang Gung University, Taoyuan 33302, Taiwan; 3 Department of Ophthalmology, Chang Gung Memorial Hospital, Linkou Branch, Taoyuan 33304, Taiwan; yihshiou.hwang@gmail.com; 4 Graduate Institute of Medical Mechatronics, College of Engineering, Chang Gung University, Taoyuan 33302, Taiwan; pc5380@hotmail.com; 5 Department of Mechanical Engineering, College of Engineering, Chang Gung University, Taoyuan 33302, Taiwan; 6 Department of Radiation Oncology, Chang Gung Memorial Hospital, Linkou Branch, Taoyuan 33304, Taiwan; 7 Graduate Institute of Clinical Medical Sciences, College of Medicine, Chang Gung University, Taoyuan 33302, Taiwan

**Keywords:** silver ink, PDMS, cell, stimulation, recording, flexible

## Abstract

This study presents a method of producing flexible electrodes for potentially simultaneously stimulating and measuring cellular signals in retinal cells. Currently, most multi-electrode applications rely primarily on etching, but the metals involved have a certain degree of brittleness, leaving them prone to cracking under prolonged pressure. This study proposes using silver chloride ink as a conductive metal, and polydimethysiloxane (PDMS) as the substrate to provide electrodes with an increased degree of flexibility to allow them to bend. This structure is divided into the electrode layer made of PDMS and silver chloride ink, and a PDMS film coating layer. PDMS can be mixed in different proportions to modify the degree of rigidity. The proposed method involved three steps. The first segment entailed the manufacturing of the electrode, using silver chloride ink as the conductive material, and using computer software to define the electrode size and micro-engraving mechanisms to produce the electrode pattern. The resulting uniform PDMS pattern was then baked onto the model, and the flow channel was filled with the conductive material before air drying to produce the required electrode. In the second stage, we tested the electrode, using an impedance analyzer to measure electrode cyclic voltammetry and impedance. In the third phase, mechanical and biocompatibility tests were conducted to determine electrode properties. This study aims to produce a flexible, non-metallic sensing electrode which fits snugly for use in a range of measurement applications.

## 1. Introduction

In recent years, a rapid increase of research interest in using cell mass measurements as a means of biomedical analysis has been observed in a wide range of scientific fields, seeking to develop inexpensive means of rapidly manufacturing electrodes capable of detection at the micron scale [1,2]. In a detection system, the most critical component is that which comes into direct contact with the test substance, thus the performance of the detection electrode determines the overall system performance. An ideal sensing electrode requires effective stimulation or recorded results, high stability and biocompatibility, and the development of sensing electrodes has gradually advanced to satisfy these requirements.

Development of integrated systems for sensing electrodes for use in biological sciences and Micro-Electro-Mechanical Systems (MEMS) technology [3] has proceeded rapidly, and sensing electrodes are now used widely in a diverse range of forms. Sensing electrodes can be roughly divided into wafer-type and flexible electrodes. The former emphasizes large scale sample testing for use with testing equipment for simultaneous testing of multiple datasets. The latter is used to conduct contact testing without damaging the measured body. Early electrodes used rigid substrates [4], but a range of different electrodes have been developed to accommodate the complexity of current testing specimens. Electrodes are used at the system front-end to make direct contact with the specimen to be measured, allowing the system back-end to easily obtain measurement parameters. Electrode durability is largely dependent on operational usage, thus maintaining electrodes in their normal working state requires use of materials which remain usable over time in regular exposure to a range of potentially damaging substances including electrolytes, acids, bases, and corrosive materials. In addition, material biocompatibility is an important consideration, and the electrode material must avoid releasing toxic substances or degrading under use [5]. Many new methods have been developed for manufacturing electrodes. Current electrode machining processes primarily use inert metals, including platinum, gold, iridium oxide, etc., as the main conductive material [6,7,8]. These materials have good biocompatibility, and have many applications in the production of micro-electrodes, but such devices are vulnerable to oxidation and corrosion over long-term usage. Recently, a rapid evolution of manufacturing flexible sensing electrodes has been noticed. As mentioned in the previous section, although metals are widely applied in sensing electrodes, they have not been widely used in flexible sensing electrodes because the expansion coefficient between materials causes them to easily crack during manufacturing which negatively affects conductivity [9]. Thus researchers have begun to search for alternative conductive materials such as carbon nanotubes, silver chloride ink, etc. [10,11,12,13,14], and non-brittle materials or flexible substrate materials such as PET, polyimide (PI), and polydimethylsiloxane (PDMS) [15,16,17] to address these problems while improving biocompatibility, flexibility and endurance.

This study aimed to develop an electrode using a flexible non-metallic material which can be used to stimulate cells during recording. Since the electrode is in contact with cells for long durations, excluding biological toxicity and maximizing electrode durability are key considerations to minimize harm to the cell. Compared to conventional electrodes, the proposed electrode is characterized by low-cost manufacturing, a simple structure and high flexibility. It uses PDMS as a substrate, and the electrode pattern etched on the substrate is bonded to the conductive material, thus reducing production time. PDMS is used to achieve high plasticity and softness, making the electrode resistant to cracking during use, thus the proposed sensing electrode can be used for simultaneous cell stimulation and recording, allowing it to be used in a wide range of tests on different surfaces.

## 2. Literature Review

A flexible electrode is defined as one which can deform under the application of force without compromising the electrode's function. In many studies, electrodes are frequently made of glass, silicon substrates, polymers and other substances. In recent years, additional interest has focused on the use of flexible materials. Compared to common substrate materials, flexible materials are inexpensive, light and plastic, allowing electrodes to measure objects with different surface geometries. This section presents a literature review of the use of flexible conductive materials in the production of electrodes.

With the maturation of MEMS technologies, sensing electrodes have been produced with a variety of metal materials including platinum, gold and iridium oxide [6,8]. These materials are now used in a wide range of electrodes to significantly enhance the range of potential applications. However, the greatest considerations for sensing electrodes are production cost and sensitivity. Thus, despite the high sensitivity of these metal-based electrodes, their production costs are prohibitively high. Therefore, in recent years, many researchers have focused on the development of new electrically conductive materials to enable the development of flexible, non-metallic electrodes. To optimize conductive performance and ensure safe sensing operations, researchers have mainly focused on maximizing durability and biocompatibility of materials for use in electrical stimulation and measurement recording. This study presents an overview of the research literature on the use of flexible, non-metallic materials in electrodes commonly used for stimulation.

### 2.1. Non-Metallic Electrodes

A board range of experiments on non-metallic electrodes was reported in the past few years. Metallic materials can have an impact on cell properties in terms of biocompatibility and cytotoxicity. Many studies have examined biological sensing technologies. In 2012, Zhu et al. [18] used polyimide (PI) to form a carbide film as a conductive material. A yellow light polyimide pattern process was used to form the electrode wire, followed by 900 °C high-temperature processing to carbonize the PI. During the subsequent yellow light process, the etching produced a flexible carbon-neural microelectrode array. This study used a single material to produce the electrode, thus reducing material requirements. The use of flexible neural microelectrode films significantly improved electrochemical stability, as opposed to traditional flexible neural microelectrodes made with electrically conductive metal. Also, Da Silva et al. [19] developed low-cost inkjet-printed electrodes using Ag/AgCl as the conductive material, printed on flexible paper and PET, thus increasing stability and durability, with results showing continuous electrochemical measurements over 30 min. Given relatively stable storage conditions, the electrodes could be reliably used for measurements after 30 days of usage. This material lends itself to the large scale production and design of a range of electrodes. In current research, Ag/AgCl is the most commonly used material for reference electrodes, providing good safety, good stability and ease of production.

### 2.2. Flexible Electrodes

In flexible electrodes, degree of flexibility is a key consideration in determining electrode performance. Generally speaking, impedance changes with the cross-sectional area and length. Applying an external force to the electrode also changes the deflection. In terms of the electrode's impedance, the chemical characteristics of electrical conductivity also change, raising the need to explore technical solutions to optimize flexibility so as to avoid impacting electrode performance. In 2009, researchers used PDMS as a substrate to produce a geometric microelectrode array, using yellow light vapor deposition to deposit a gold electrode on the PDMS substrate. They then used yellow light fabrication processes to produce a microelectrode array with a three-tier structure [17]. This array featured a depressed electrode, producing a uniform current density during the stimulation process. The size of the current density impacts the electrode's corrosion rate, and thus potentially produces a tissue-damaging stimulus. In addition, Chou et al. [20] developed a deformable electrode array based on a PDMS substrate. The polymer parylene was used as a metal cladding layer, while the electrode structure on the PDMS substrate was coated with a layer of parylene. Vapor deposition was used to deposit the metal, after which yellow light etching was used to form the electrode. The uppermost layer was then coated with paraylene to produce a four-layer metal electrode, in which the parylene helps prevent cracking during the production process.

### 2.3. Stimulation Applications of Electrodes

Electrical stimulation is a promising direction in technology development, with many potential uses such as the restoration of physiological functions and the induction of hyperpasia in tissue. Stimulating electrodes require sufficient charge injection to induce an arousal response, while minimizing tissue damage to the contact site. Therefore, the choice of electrode material is a key consideration, and the stimulation function is determined by the charge storage capacity (CSC) [21]. Thus, an electrode material with a high CSC allows for electrode miniaturization, producing higher current densities and thus allowing for operation within a safe voltage range to avoid electrolysis and reducing damage to the stimulation site. In 2012, Negi et al. [22] developed a nerve electrode array, using iridium oxide (IrOx) for the electrode surface to fabricate a film layer. They produced two types of films: a sputtered iridium oxide film (SIROF) and an activated iridium oxide film (AIROF). They deposited the electrode arrays on these films to conduct electrochemical performance assessments and to compare the applicability of electrode materials. In addition, Luo et al. [23] used composite materials to successfully fabricate a conductive coating on a micro-electrode array. They used a polymer nanomaterial (3,4-ethylenedioxythiophene) (PEDOT) mixed with carbon nanotubes as a conductive material which was deposited on the platinum electrode. Electrochemical measurements showed that the PEDOT/CNT composite had excellent stability, without cracks or delamination, and resulted in a significantly improved charge injection capability.

This section discusses research directions in flexible materials, non-metallic electrodes and stimulation electrodes. The review of the literature revealed that most sensing electrodes use metallic materials, and that technologies for such metallic electrodes for measurements are quite mature. However, sensing electrodes are manufactured using semiconductor production processes which are characterized by high complexity and cost. Therefore, many researchers have sought to discover alternative conductive materials, including conductive ink, to provide good electrical conductivity, high sensitivity, low cost, simplified production and high biocompatibility. Therefore this study uses Ag/AgCl as a conductive material to produce electrodes. The resulting electrodes not only provide good detection levels, but have no impact on successive experiments. PDMS features high biocompatibility, structural stability and plasticity and the production of electrodes based on PDMS is relatively simple and quick. Therefore, this study proposes developing a PDMS substrate to produce a flexible, non-metallic electrode using Ag/AgCl. The proposed method can be used to create a fully-formed sensing electrode which can be applied to a wide range of surfaces for various tests.

## 3. Characteristics of PDMS Material

**Figure 1:**
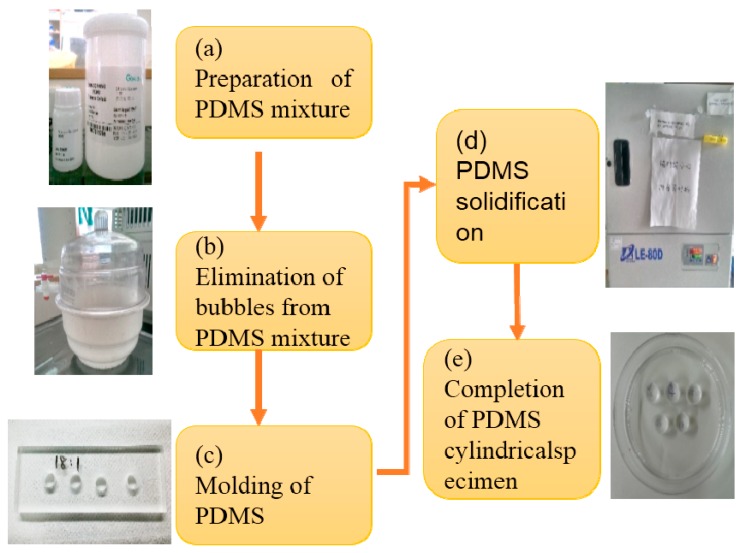
Production process of PDMS cylindrical specimen; (**a**) PDMS mixture was prepared by mixing PDMS pre-polymer and curing agent; (**b**) Bubbles were eliminated from PDMS mixture by vacuum pumping; (**c**) PDMS mixture was poured into the PMMA mold; (**d**) PDMS solidified by heating in oven at 70 °C for 1 h; (**e**) PDMS cylindrical specimen was removed from the PMMA mold.

The characteristics of PDMS material were analyzed using a tensile testing machine. Deformation of PDMS specimens at different PDMS mixing ratios was examined under pressure. We describe the experimental design and procedure for manufacturing PDMS columns with different mixing ratios, followed by using the tensile testing machine (JSV-H1000, Japan Instrumentation System, Sakurai-shi, Japan) to quantify the compression results for different PDMS ratios. This study aims at designing a flexible electrode made of highly biocompatible PDMS (Sylgard^®^ 184, Dow Corning, Auburn, MI, USA) for use on biological specimens. The Young's modulus of PDMS is 0.4~1.0 MPa, which is close to that of biological tissue. The high plasticity of the PDMS allows it to be adapted to biological tissues in various shapes, allowing the resulting electrode to bend and fit closely against the specimen [24]. Lei et al. [25] examined the mechanical properties of PDMS with different mixing ratios. Based on the results, the flexibility experimental analysis test for PDMS conducted in the present study was based on five different mixing ratios by weight as follows: 12:1, 14:1, 15:1, 16:1 and 18:1. The completed PDMS solution was mixed into a self-manufactured cylindrical mold. Liquid was discharged through air vacuuming, and the mold was then placed in a convection oven to cure for 1 h at 70 °C to produce a cylindrical specimen with a diameter of 12 mm, a height of 10 mm and a volume of 1.13 cm^3^. The production process is shown in Figure 1.

**Figure 2:**
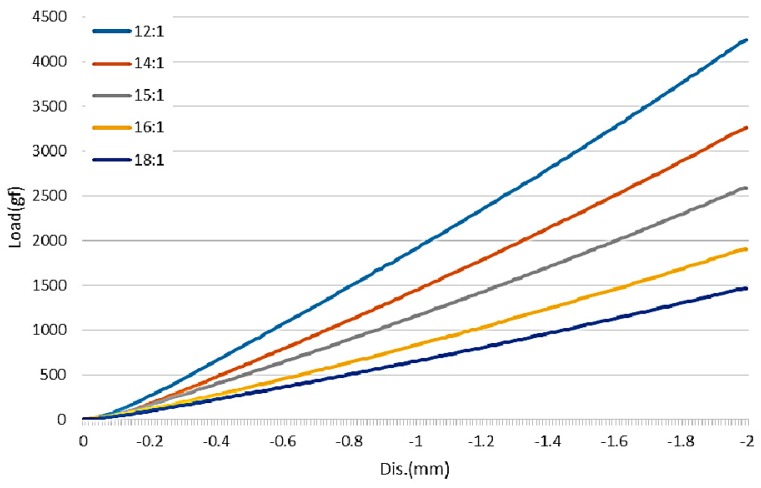
Results of stress-strain analysis of PDMS materials in different weight ratios.

A tensile testing machine was used to perform the stress and strain analysis of the PDMS material. Different weight ratios of PDMS result in different resistances to stress and strain. PDMS specimens were tested in a limited compression range at a constant rate of movement, observing the changes in pressure for each weight ratio. The experimental parameters involved a compression of 2 mm and a rate of motion of 1 mm/min. Test results are shown in Figure 2. The weight ratio of 12:1 produced the maximum slope (stress/strain) indicating that this PDMS ratio is relatively less easily compressed, while the 18:1 weight ratio had the smallest slope (stress/strain), indicating that this PDMS ratio is more easily compressed. Thus, the experimental results showed a smaller PDMS weight ratio increase rigidity and reduced compressibility, but was better able to withstand maximum pressure. Stress-strain analysis results showed that the 12:1 ratio had better strength, while 15:1 was weaker than 12:1 and 14:1 with better ductility. Although the 16:1 and 18:1 ratios had better ductility, their overall strength was poor, raising concerns about potential electrode breakage. Therefore, the 15:1 weight ratio was selected in the present study for subsequent experiments.

## 4. Materials and Methods

### 4.1. Experimental Materials and Equipment

Materials and equipment used in this study were PDMS (Sylgard^®^ 184, Dow Corning, Auburn, MI, USA), Silver Chloride Ink (AGCL-675, Conductive Compounds, Hudson, NH, USA), Sterile solution (Alcon, Fort Worth, TX, USA), tensile testing machine (JSV-H1000, Japan Instrumentation System, Sakurai-shi, Japan), CNC Engraver (EGX-400, Roland, Japan), plasma wafer bonding machine (Plasma cleaner, Harrick Plasma, Ithaca, NY, USA), Impedance Analyzer (Versa STAT4, Princeton Applied Research, Oak Ridge, TN, USA), and spin coater (TAA-00053, Pentad Scientific, Hsin-Chu City, Taiwan). In the electrode production process, materials were washed with deionized water, and all production took place at room temperature (25 °C).

### 4.2. Design of the Flexible Electrodes

**Figure 3:**
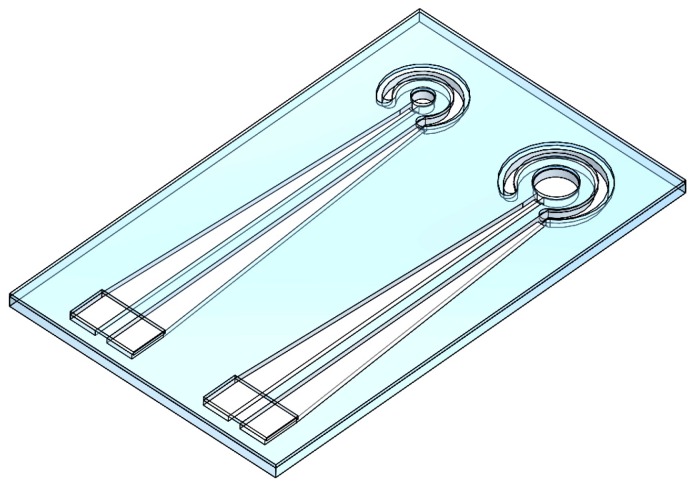
Conceptual drawing of the flexible electrode.

The flexible electrodes were fabricated by using microfluidic channel technology with a replica molding. The mold was made of poly(methyl methacrylate) (PMMA) and produced by using a CNC engraver. The electrode layer defined the electrode size with conductive silver paste used to provide an increased adhesion area, thus increasing the current storage capacity. As previously noted, the relevant literature largely overlooked conductive materials. Thus a relatively large working electrode was needed to observe the impact of electrode flexibility on stimulating current. To explore the impact of the working electrode surface area on stimulation current, this study produced electrodes with working areas of 0.785 mm^2^ and 3.14 mm^2^. The working electrode was surrounded by the auxiliary electrode to form a flexible electrode that can both receive signals and stimulate (length: 23 mm, electrode spacing: 0.7 mm, height: 0.5 mm). The channel was filled with conductive silver paste as the conductive material for the flexible electrode, consisting of a two-layer structure of PDMS material. The top layer covered the electrode lead to prevent exposed wires from affecting measurement results. An impedance analyzer was included in the electrode terminal to observe and record physiological changes during cell stimulation as the current creates a circuit within the electrode. The flexible electrode is illustrated in Figure 3.

### 4.3. Manufacture of the Flexible Electrodes

In this study, the fabrication of flexible electrodes required several key processes, including soft lithography, silver chloride ink injection in the electrode layer, and PDMS bonding of electrode/coating layer. The electrode layer was fabricated by using soft lithography. The material was PDMS at a 15:1 weight ratio of PDMS pre-polymer and curing agent. The PDMS mixture was placed in a vacuum chamber which was evacuated until all bubbles in the mixture were eliminated. It was then poured into a PMMA mold produced by using a micro-engraver. Then, the mold was then placed in the spin coater at a speed of 500 rpm for 20 s. Next, PDMS was baked for 1 h, and removed from the mold. Silver chloride ink was then injected into the channels of the electrode layer to ensure that the current for cell stimulation and recording can be supplied and received without the electrode cracking as it bends. The PDMS bonding of the electrode/coating layers covered the silver chloride ink with PDMS to insulate the wire. PDMS thickness was controlled to produce a thin film which does not restrict the bending of the electrode.

**Figure 4:**
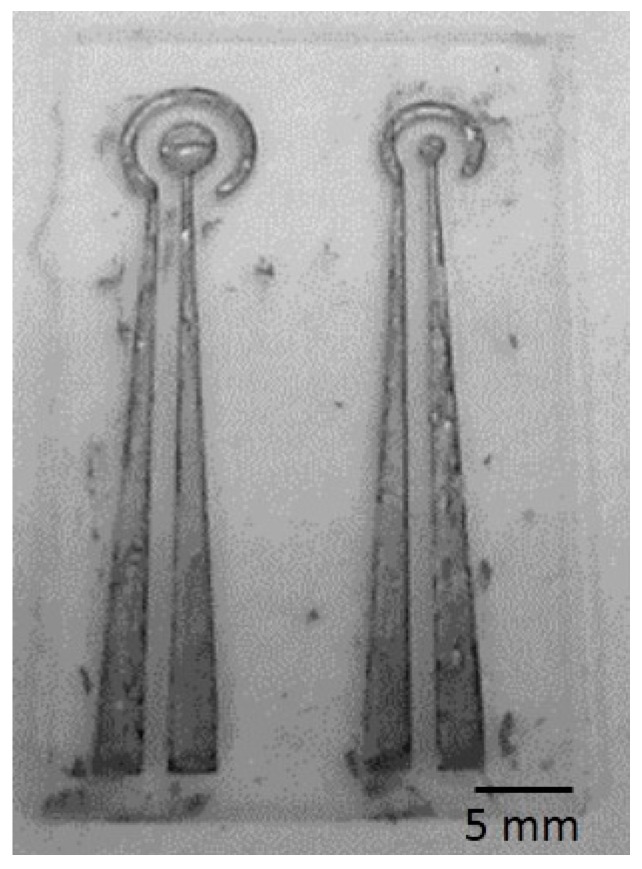
Photograph of the flexible electrodes.

Silver chloride ink was used in this study. It is a biocompatible adhesive as the conductive material for the flexible electrode. The conductive material should be evenly injected into the recess of the electrode layer. Silver chloride ink is highly viscous, thus to ensure an even injection, it must be diluted to a viscosity by mixing with an organic solvent such as methanol at a ratio of 1:1 through stirring in an ultrasonic vibrator for 30 min. It was then injected into microfluidic channel of the PDMS electrode layer. Excess silver chloride ink was scraped off, and the electrode was air-dried overnight. The finished device is shown in Figure 4.

The dimension of the flexible electrodes was defined by micro-engraver machine. The resulting process was simple and had a high success rate in producing flexible electrodes which were resistant to cracking. Most previous researches on the production of flexible electrodes directly deposited PDMS through vapor deposition or sputtering on the electrode material [26,27], but these techniques were likely to result in cracking because while PDMS was an elastic material, its expansion coefficient was considerably different from that of metal materials, thus the deformation of the metal surface can easily produce cracks, thus impacting the electrode's continuity. Scaling up production may further exacerbate these problems, resulting in poor yields. Using micro-engraving techniques to produce flexible electrodes was relatively more straightforward and had higher yields. Thus the proposed process has the advantages of simplicity and high yield rates.

### 4.4. Electrochemical Properties

Electrochemical impedance spectroscopy (EIS) is an important electrochemical testing technique [28] which uses very small exchanges of sine wave signals to detect the resistance value between two electrodes, usually within a peak-to-peak range of 5~10 mV. This method provides highly sensitive, low cost, simple testing processes and can be used directly in gasses and liquids. Due to these and other advantages, EIS has emerged as an important tool for detecting electrochemical properties for decades. Electrochemical impedance analysis generally uses fixed voltage sweep measurements, primarily for electrodes with very small AC signals because the sensing electrode theoretically does not produce unnecessary current, thus the electrode reaction is not apparent in the measurement process, and making EIS a more attractive testing method.

Cyclic voltammetry (CV) is a widely used electrochemical analysis method. Its advantage is that the electrolytic method used for chemical analysis can exhibit electrochemical characteristics, and control their potential to detect electrochemical reactions [29,30]. CV operates in a limited potential range, and is a way of applying cyclical potential. From the outset, the potential is applied at a fixed rate to the final potential, and then the same rate of change is used to revert to the initial potential to form one cycle. The potential sweeps back and forth to obtain a current-potential diagram which can be used for further electrochemical investigations.

The electrochemical properties of an electrode are key indicators for stimulus effect and signal recording. A good electrode has high stability, low impedance and a high charge storage capacity. In this study, an impedance analyzer was used to conduct electrochemical impedance spectroscopy and CV analysis. A sterile buffered solution was used for the measurement. An AC electric field of 10 mV with the scanning frequencies range from 1 kHz to 100 kHz was used to measure the current impedance and electrochemical detection response at different frequencies.

### 4.5. Biocompatibility of the Electrode

To create a flexible electrode for cell stimulus and recording that can be used safely over long durations, the electrode must have a high degree of biocompatibility and non-cytotoxicity. Some biological materials contain soluble substances which can cause inflammation and tissue reaction. In this study, test of lactic acid production was used to evaluate electrode biocompatibility. Animals produce energy through sub-aerobic and anaerobic respiration. Through anaerobic respiration, cells produce lactic acid. Lactate molecular (formula C_3_H_6_O_6_, Mohr mass of 89.07 g/mol) is an aqueous solution of lactic acid which releases protons to produce lactic acid ions. Bodies produce energy mainly in the form of glucose. In both aerobic and anaerobic respiration, glucose transporter 4 moves glucose into the cell where glycolysis produces pyruvate in a precursor to the production of energy. Given sufficient levels of oxygen, the glycolysis process can be split into three steps: First, glucose is converted into pyruvate, which is then introduced to the mitochondria through the TCA cycle to produce NADH and FADH2. Because oxygen is added in this step, the energy obtained is referred to as aerobic respiration, which is the main means by which organisms produce energy. Through aerobic respiration a single glucose molecule will produce 38 adenosine triphosphate (ATP) molecules [31]. However, in different environments, cells produce energy in different ways. Aerobic respiration takes a relatively long time to take effect, thus when an organism has insufficient oxygen or engages in intense exertion over a short time period, pyruvic acid with hydrogen is used to produce two ATP molecules [32], a process called anaerobic respiration. Previous studies have found that cancer cells tend mostly to derive energy from anaerobic respiration, and the normal lactic acid content in blood averages 1–2 mmol/L. Studies have shown that cultures of different cancer lines and cancer cells can secrete lactic acid to the supernatant and, when the supernatant is cultured for several days, its lactic acid content and cell count show a significant and positive correlation [33].

In this study, tests of biocompatibility was performed by immersing the flexible electrodes in petri dishes containing nasopharyngeal carcinoma cells (cell line: BM1). After collecting 100 μL of cell culture supernatant at 0, 24 and 48 h, it was centrifuged to remove the culture medium from the lactic dehydrogenase. A lactate assay kit (MAK065, Sigma, St. Louis, MO, USA) was used for subsequent analysis of the lactic acid.

## 5. Results and Discussions

### 5.1. Electrochemical Properties of the Flexible Electrodes

**Figure 5:**
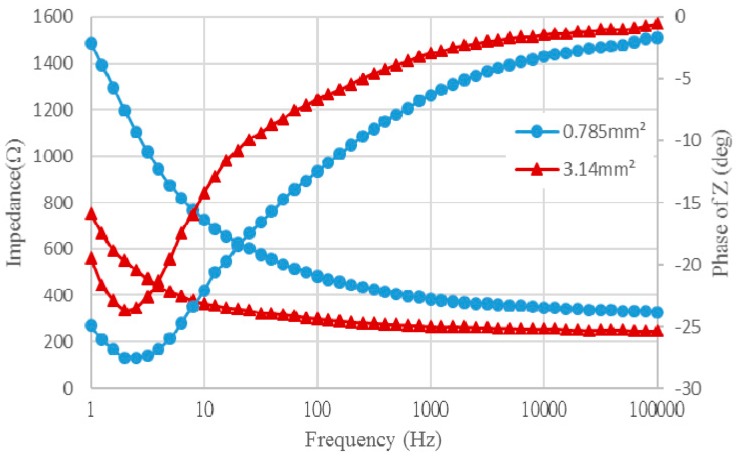
Impedance, phase angle and frequency of electrodes.

To validate the utility and stability of the developed flexible electrode, an impedance analyzer was used to measure the change in impedance at different frequencies. Figure 5 shows the testing equipment setup, where a sterile buffered solution (0.74% NaCl) was used for testing. The solution was dropped onto the electrode's detection region. The conductive solution created a circuit between the flexible electrode's working surface and the reference electrode, allowing to the changes in the resistance value to be measured. The impedance-frequency relationship of electrode is shown in Figure 5.

**Figure 6:**
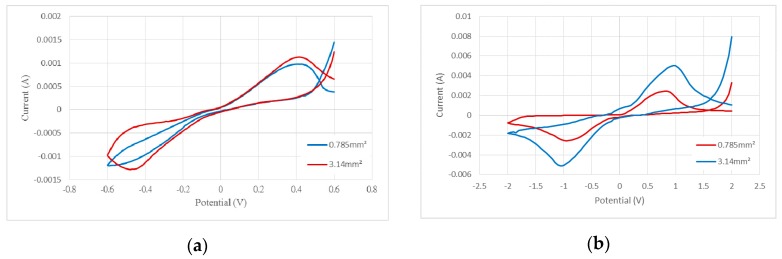
Cyclic voltammetry curves for different electrode surface areas with a voltage range of (**a**) ±0.6 V and (**b**) ±2 V.

With sweeping frequency from low to high, the impedance of the electrode under frequency changes of the AC signal was recorded. In this study, the working surface areas of two flexible electrodes were 0.785 mm^2^ and 3.14 mm^2^, respectively. Following quantitative impedance analysis, we compared the impedances of the two different electrode surface areas at a frequency of 1 kHz. The impedance ratio was about 1.45, mostly due to the relationship between electrode surface area and resistance. Under the same circumstances, as the electrode surface area shrinks, the resistance increases. Thus, while electrodes with increase surface area are easier to design and produce, they fall far short in terms of practical utility. At 1 kHz, the impedance values for the electrodes of 0.785 mm^2^ and 3.14 mm^2^ were about 385.5 Ω and 265.7 Ω, respectively. Because the electrochemical properties determine the effectiveness of the sensing electrode, the key factor influencing the electrode's stimulus effect is the CSC. An electrode with a high CSC can produce effective stimulation in a safe voltage and current range. Usually, CSC is determined by CV based on measurements taken with an impedance analyzer to determine the voltage range and scan rate. We set the scan rate as 10 mV/s and 50 mV/s for the voltage ranges of ±0.6 V and ±2 V, respectively. Figure 6 shows the CV graph for the measurement results. To calculate CSC, the following formula is used:

$$\mathrm{CSC}=\frac{1}{\nu A}\int_{Ec}^{Ea}\left|i\right|\mathrm{dE}\left(C/{\mathrm{cm}}^{2}\right)$$

where *E* is the electrode potential, *i* is the measured current (AMP), *E_a_* and *E_c_* are the anodic and cathodic potential limits (V), respectively, *A* is the geometric area of the electrode, and *ν* is the scan rate. The results were summarized in Table 1. The calculated capacity was used to define the maximum durable current range of the flexible electrode.

To test whether stimulation of the subretinal space could restore retinal function, Chow et al. [34] implanted a bag-shaped electrode to stimulate the visual cortex. The electrode's surface area was 3.6 mm^2^ [35]. The CSC could be reduced as far as 2.8 nC/cm^2^. This showed that different electrode surface area measurements and materials affected stimulus performance. The electrode designed in this study was larger than that in previous studies, but in terms of stimulation performance, the proposed electrode had a better CSC, indicating that the proposed flexible, non-metallic electrode provided a better stimulation effect.

Electrochemical test results showed that using silver chloride ink as the conductive material in flexible electrodes resulted in increased stability, while the increased CSC also increased stimulation, thus enhancing electrode safety and durability. In electrochemical impedance tests, changes to impedance can be used to determine the merits of the electrode recording signal in that such changes can determine whether the signal during recording is impacted by external interference, and whether electrode durability is impacting signal recording. The signal received by the electrode is feedback following cell stimulation, which serves to verify the stimulus function. In the measurement process, a BBS sterile solution (physiological saline solution) is used as the conductive solution for testing the electrode. A typical living organism consists of innumerable substances, such as red blood cells, serum proteins, carbohydrates, etc. Therefore, the use of a sterile buffer solution only serves as a reference for research. We hope that, in the future, the electrodes can be applied in vivo study to obtain accurate measurement results.

### 5.2. Mechanical Properties of the Flexible Electrode

**Figure 7:**
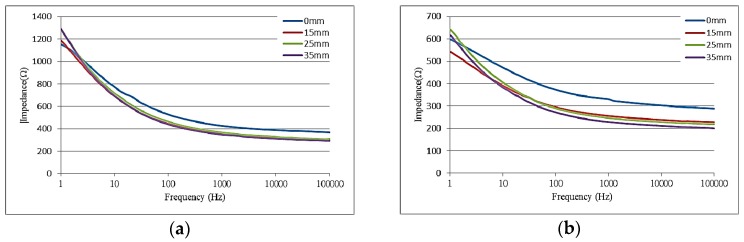
Relationship of impedance and frequency for different arc radii curvatures. (**a**) Electrode area 0.785 mm^2^; (**b**) Electrode area 3.14 mm^2^.

In this study, a soft, flexible electrode was developed which was kept stationary while working in a biological environment. Results shown in Figure 7 indicate the impact of flexibility on the impedance of the as-produced soft, flexible electrode. Electrodes were produced with arc radii of 15, 25 and 35 mm, and a plane surface of 0 mm. Measurements were taken for impedance changes on different surfaces. Results showed that impedance found no significant difference with different arc radii. As the electrode bent, the wire underwent a greater deformation. Without affecting or damaging the electrode, bending of the electrode was unlikely to affect the surface impedance.

**Figure 8:**
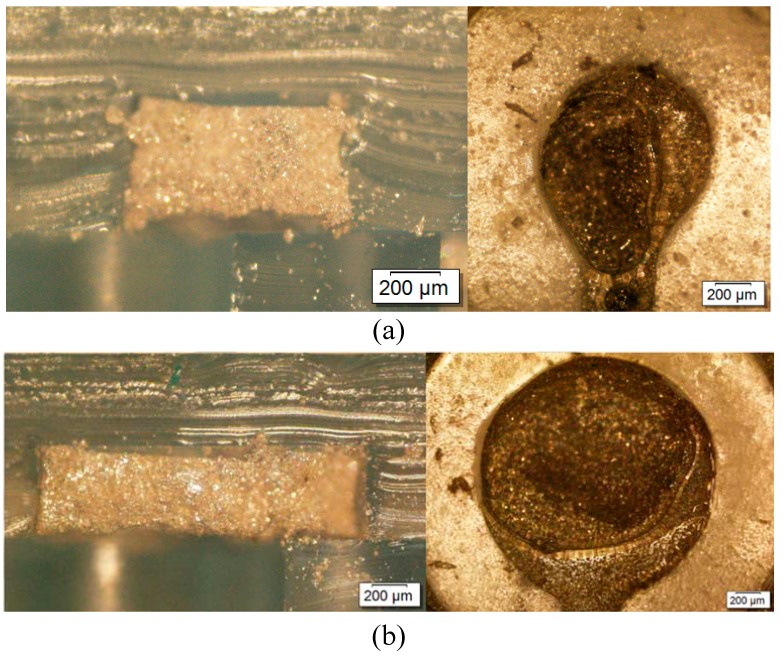
Cross-sectional and frontal views of electrode with a surface area of (**a**) 0.785 mm^2^ and (**b**) 3.14 mm^2^.

Moreover, microscopic observation of the frontal and sectional views of the electrodes were investigated by an upright microscope. The observed results are shown in Figure 8. The microscopic images showed that the surface area of the completed electrode was 0.8 times the original size, and was 0.2 times the original height. The difference in size between the mold and the electrode indicated that the mold size should be modified to maximize the precision of the electrode size.

### 5.3. Biocompatibility of the Flexible Electrode

**Figure 9:**
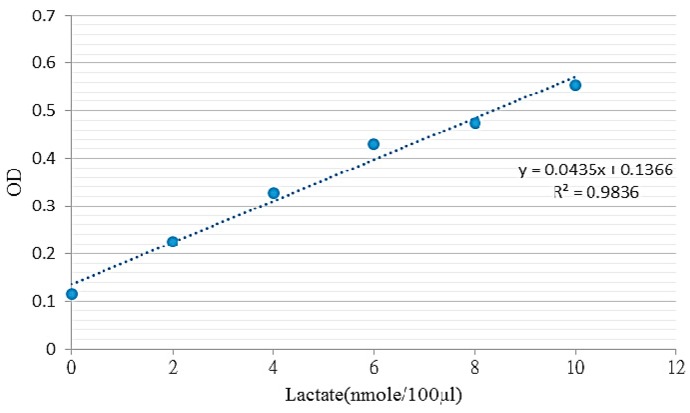
Relationship between lactic acid standard concentration and OD value.

**Figure 10:**
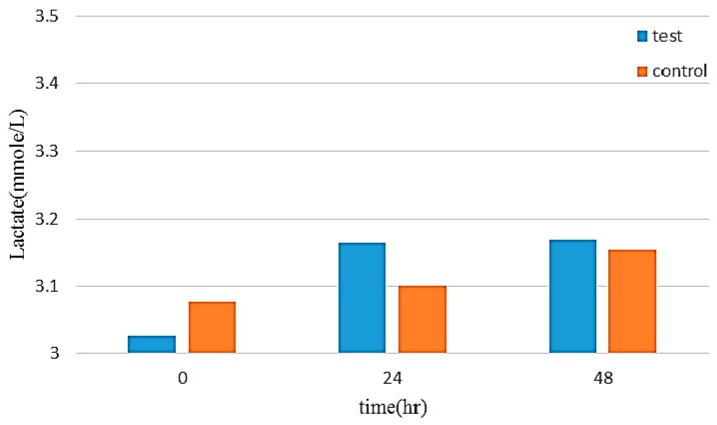
Experimental comparison group lactic acid content.

The production of lactic acid in the cell metabolism can be used as indicators for cell growth. The lactic acid color comparison test results can be used to obtain standards for concentration and absorbance (OD), which are then used to calculate a regression curve to the introduction of the lactic acid content, with results shown in Figure 9. Then, the supernatant of culture medium was collected at 0, 24, and 48 h. Over extended testing, the lactic acid content of the experimental group and the control group showed that the flexible electrodes exhibited no significant cell toxicity (Figure 10).

## 6. Conclusions

This study presents a flexible electrode fabricated by using silver chloride ink as the conductive material. Micro-channel techniques were used to produce a conductive layer and a coating layer which were then bonded to produce a completed flexible electrode. This method only required silver chloride ink and PDMS, thus reducing sample and material consumption. Compared to conventional electrode manufacturing processes, the proposed electrodes were produced using sputtering and microchannel techniques, thus simplifying the overall process. Observation and analysis were used to determine the stress-strain relationship for different weight ratios to identify an appropriate mixing ratio for the flexible electrodes. Electrochemical testing results showed that the use of silver chloride ink resulted in increased stability, and the CSC also produced enhanced stimulation, thus enhancing the safety and durability of the flexible electrodes. Lactic acid tests in cell cultures also showed the flexible electrodes had good biocompatibility and are suitable for observation and use. Future work will focus on further refining the electrode structure and further increasing the sensing area to produce a flexible electrode with minimal impedance in a small space for a wide range of measurement applications.

## Figures and Tables

**Table 1:** Calculated charge storage capacity (mC/cm^2^) under different conditions.

Voltage Range/Area	0.785 mm^2^	3.14 mm^2^
±0.6 V	138.16	61.57
±2 V	86.98	54.14
